# Effects of bleeding of *Actinidia arguta* (Sieb. & Zucc) Planch. ex miq. on its plant growth, physiological characteristics and fruit quality

**DOI:** 10.1186/s12870-023-04560-w

**Published:** 2023-11-02

**Authors:** Yaxuan Jiang, Pei Lei, Le Ma, Kun Dong, Yu Zhang, Jia Zhao, Xinyu Guo, Jianxin Liu, Wei Li, Lei Tao, Fanjuan Meng

**Affiliations:** 1 College of Life Science, Northeast Forestry University, Harbin, 150040 China; 2 Horticultural Sub-Academy, Heilongjiang Academy of Agricultural Sciences, Harbin, 150069 China; 3 Forest Botanical Garden of Heilongjiang Province, Harbin, 150040 China; 4 Harbin Engineering University, Harbin, 150001 China; 5 Crop Tillage and Cultivation, Institute of Heilongjiang Academy of Agricultural Sciences, Harbin, 150086 China

**Keywords:** *Actinidia arguta* Sieb. & Zucc. Planch. ex Miq., Bleeding, Fruit quality, Physiological characteristics

## Abstract

Bleeding is as particularly a serious phenomenon in *Actinidia arguta* and has important effects on this plant’s growth and development. Here we used *A*. *arguta* to study the effects of bleeding on the growth and development of leaves and fruits after a bleeding episode. We detect and analyze physiological indices of leaves and fruit after bleeding. The result revealed that the relative electrical conductivity and malondialdehyde (MDA) of leaves increased in treatment. Nitro blue tetrazolium chloride (NBT) and 3,3-diaminobenzidine (DAB) staining revealed the accumulation of reactive oxygen species (ROS) in leaves after bleeding. The chlorophyll content and photosynthetic parameter of plants were also decreased. In fruits, pulp and seed water content decreased after the damage, as did fruit vitamin C (Vc), soluble sugar content, and soluble solids content (SSC); the titratable acid content did not change significantly. We therefore conclude that bleeding affects the physiological indices of *A*. *arguta*. Our study provides a theoretical basis for understanding the physiological changes of *A*. *arguta* after bleeding episodes and laying a timely foundation for advancing research on *A. arguta* bleeding and long-term field studies should be executed in order to gain insights into underlying mechanisms.

**Supplementary Information:**

The online version contains supplementary material available at 10.1186/s12870-023-04560-w.

## Introduction

*Actinidia arguta* Sieb. & Zucc. Planch. ex Miq., a plant widely distributed in East Asia including to China, Korea, and Japan, has gradually developed into the second largest *A*. *arguta* species in the world [1–3]. It has excellent traits, such as cold tolerance, delicious fruit, and immense health benefits [4, 5]. Additionally, its edible fruit with a distinctive flavor has a high content of vitamin C, abundant nutritious substances as well as medicinal uses; therefore, it is considered as one of the most nutritious fruits [6–8]. In particular, given its edible peel and bright fruit coloring, the majority of consumers recognize this fruit and its popularity is rising because of people’s greater awareness of the health benefits with eating *A*. *arguta* [9]. Furthermore, due to its high frost tolerance, this plant also has many horticultural advantages over the common *A*. *arguta* [10], yet many plants have the phenomenon of bleeding.

Bleeding in plants is actually a very common phenomenon. In general, bleeding is described by guttation from or wounding to leaves, stems and roots for xylem pressure. However, the mechanism by which bleeding affects plant physiology and fitness still remain largely unknown [11]. Bleeding can occur in any season and under any environmental regardless of plant age and the position of the wounded branch. Typically, the length and position of branches is directly related to the intensity of bleeding [12]. Therefore, the factors influencing the occurrence of bleeding include season, species, temperature and soil temperature [13–15]. A work use the bleeding sap to investigate phloem loss-of-function in the context of leaf physiological processes, the mechanisms of phloem turgor maintenance under drought [16]. Other work has shown that MeSWEETs genes can predominately transfer sucrose via bleeding saps in plants to foster resistance to water deficit and salt stress by modulating their sugar distribution [17].

Recent studies have also suggested that bleeding can lead to decayed of bud burst, flowering set, and flower bud formation [18]. In addition, bleeding can lead to nutrient losses and is capable of inducing the onset of plant bleeding sap canker disease [19, 20]. Intriguingly, there are no obvious plant fitness benefits to bleeding on a branch; however, bleeding sap contains a large number of beneficial substances, so bleeding sap is widely collected and used as medical product [21, 22]. Many plant species can secretion bleeding sap after bleeding. For example, grapevine’s bleeding sap can be used as a medical health product and to improve fruit quality as a growth regulator [23, 24]. Root-bleeding sap is a sign of excess root pressure, plant growth potential, and root activity [25]. Other work reported that luffa’s bleeding sap can improve the antioxidant capacity of mice [26]. And bleeding sap from bitter gourd bleeding sap can improve both the liver and kidney functioning and the immune function of diabetic mice [27, 28]. Hence, studied the bleeding sap mechanism of one or more species can provide useful information for keeping their fruit fresh and promoting their plant growth.

As woody plant species that can bleed due to root pressure [29], *A*. *arguta* stem can generally present bleeding after they are cut or when the temperature changes drastically. In just a short time, much xylem sap can bleed out (more than 100 mL·h^− 1^) [30]. Because xylem sap is rich in nutrients, the loss of this sap may impair growth and fruiting. In other words, bleeding often occurs for commercial *A*. *arguta* plants at any time, from to early spring to late spring. Due to the large pith core of its branches, *A*. *arguta* is susceptible to freezing damage [31]. The cold spell in later spring mainly occurs in April and May, when the temperature normally rises but then sharply drops, resulting in freezing damage to *A*. *arguta*. At this time, *A*. *arguta* has germination, so the freezing damage is more serious [32]. When the temperature becomes too low, within the unbearable range of plants, it may hinder their growth of plants and even lead to their death of plants [33]. When the frost damage is severe and the epidermis is broken in plants at 1 m above the ground, then the liquid flows out during the bleeding episode [34].

The bleeding in *A*. *arguta* is a serious problem in the face of environmental changes and will likely cause dramatic yield losses and a deterioration of its fruit quality. However, few studies have investigated bleeding in *A*. *arguta*. Previous studies have demonstrated the bleeding can influence the wheat yield, plant growth and fruit quality [23, 24, 27]. Accordingly, in this study, we use the common *A. arguta* ‘Kuilv’ variety in northeast China. We designed the current experiment to study the physiological variation and structure of 1-year-old bleeding *A*. *arguta* shoots in other to examine the influence of bleeding on the fruit quality. The results revealed the changes in the phenotype and physiological indices of bleeding in *A*. *arguta* in response to bleeding. These findings provide a theoretical basis for studying the breeding and molecular mechanism of *A*. *arguta*.

## Materials and methods

### Plant materials and growing conditions

The leaves and fruits of *A*. *arguta* were collected from the annual branching ‘Kuilv’ variety at the *A*. *arguta* Base of Horticulture Branch of the Heilongjiang Academy of Agricultural Sciences. Three similar growing and marked 1-year-old shoots on different branches of 10 to 12 randomly selected *A*. *arguta* individual plants were treated by pruning. We washed and dried the *A*. *arguta* stem with distilled water near the site of pruning, and cut a part at the same position at the top. Then we affixed a glass bottle to each cut stem and tied it tightly with a sealing film to collect the bleeding sap (Fig. S1). The non-pruned trees served as the control group and the artificially pruned trees were the treatment group. The leaves and fruits of the trees in both groups were collected and immediately stored at -80 °C. The following tests described below were repeated using three replicates per treatment or control. All chemicals and materials were purchased from (Meilai Co., Harbin, Chian).

### Histological analysis of leaves

Paraffin sections of leaves were made by following the methodology of Liu et al. with minor modifications applied [35]. The leaves were fixed immediately in FAA (10% formaldehyde (37%), 5% acetic acid, 50% ethanol) for 24 h, and then placed inside a lidded glass bottle containing 70% tert-butanol-glycerol mixture (1:1, v/v), for 2 days, at 50 °C. Then, each sample was immersed in a 50% paraffin solution (tert-butanol: paraffin (Leica) = 1:1, v/v) for 6 h at 56 °C. Next, all the samples were embedded in a 75% paraffin solution (tert-butanol: paraffin = 1:3, v/v) and polymerized for 6 h at 58 °C. Finally, the paraffin was poured into the steel embedding die (21 mm × 21 mm × 5 mm) and adjust the position of the material adjusted accordingly. After cooling them at room temperature, the paraffin blocks were processed at -20 °C for 2 min, and then all embedded samples were sectioned (10 μm thickness) using a slicer (Leica RM2245) (Soly industrial Co., LTD., Shanghai, China). Each sample (from a cunt stem) was attached to a positively-charged adhesive slide (Seitai, 188,105 W). These slides were put in a 45 °C-baking machine for 50 min. Then Safranin O (0.5% [w/v]) staining was performed for 9 h, at room temperature, and images then obtain under an Olympus BX51 microscope (Fulai, Shanghai, China). Finally, the length was measured using ImageI software (National Institutes of Health, Bethesda, Maryland, USA).

### Measurements of physiological trait

The leaves of *A*. *arguta* were collected at four growth stages: initial flowering stage, blooming stage, fruiting stage, and fruit maturity stage. The leaf length, leaf width, petiole length, petiole width, leaf area and longest leaf of the plants were statistically analyzed according to the method reported by Li et al. and then photographed [36]. Relative water content (RWC) was measured as described by Huang et al. [37].

Determination of electrolyte conductivity was carried out following the method described by Sobrinho et al. [38].To do this, water was added 5 mL into a test tube, and recorded as ‘E0’ by the leaf conductivity meter (DDS-IIA, Leizi, Shanghai, China). To each test tube, a leaf sample was added 0.1 mL, and the shock was performed at 180 rpm for 1 h, and then conductivity was recorded as ‘E1’. After placing the tube in a boiling water bath for 15 min, followed by cooling, the conductivity was recorded again, as ‘E2’. Electrolyte conductivity was calculated with the formula electrolyte conductivity (µS/cm) = (E1 − E0)/(E2 − E0).

To determine the malondialdehyde (MDA) content of leaves, we followed the method of Yang et al. [39], with minor modifications. To each 0.3-mL samples, we added 2 mL 0.5% TBA buffer, and boiled this for 15 min in a water bath. After cooling, the samples were centrifugated at 12,000 rpm for 10 min. The OD_450_, OD_532_, and OD_600_ were determined for the ensuing supernatant solution s by a UV/VIS spectrophotometer (Thermo, USA).

The chlorophyll content of leaves was measured using the methodology of Huang et al., and their photosynthesis quantified as described by Jiang and Chang et al. [37, 40, 41]. The *T*r (transpiration rate), *P*n (net photosynthetic rate), *C*i (intercellular CO_2_ concentration) and *G*s (stomatal conductance) of the *A*. *arguta* leaves were measured at 11:00 a.m., using a LI-6400 portable photosynthetic system (LI-COR, Lincoln, NE, USA). Leaf portions measuring 6 cm^2^ (3 cm in length, 2 cm in width) were placed in the instrument under white light (1,000 µmol mol m^− 2^ s^− 1^). Five plants were chosen from each group and each plant was measured five times. The fifth fully-expanded leaf of each plant was selected for the chlorophyll determination. For this, one leaf was taken per plant, and 0.2 g of its fresh tissue was weighed and placed in 10 mL of an 80% acetone solution, for 10 h in the dark, to determine the OD_470_, OD_645_, and OD_663_.

### Investigation of reactive oxygen species (ROS)

Histochemical staining of O_2_^−^ and H_2_O_2_ was done using nitro blue tetrazolium chloride (NBT) and 3,3-diaminobenzidine (DAB). The leaves of *A*. *arguta* at different growth stages were immersed for 24 h in 0.5 mg·mL^− 1^ NBT solution or a 0.5 mg·mL^− 1^ DAB solution. These stained leaves were rinsed with 80% ethanol, at 70°С for 15 min, and then bleached in a boiling water bath with bleaching solution for 10 min, before the leaves were photographing them [37].

### Fruits quality analysis

Five fruits were randomly selected and each fruit weighed on a digital scale (Sartorius, Gottingen, Germany). Fruits slices were cut (each ca. 5 g) and oven-dried at 105℃ to constant temperature drying oven to constant weight (DHG-9070 A, Yiheng, Shanghai, China), and this repeated three times. The flesh consisted of the peeled tissue of the whole fruit; seeds included all those mature and immature seeds in a given fruit. Five *A*. *arguta* were randomly selected from each group and divided into two groups. Their fresh weight of whole fruit, the fresh weight of pulp and the fresh weight of seeds was successively weighed.

Vitamin C (V_C_) were measured as already described elsewhere [36]. The 100-grain weight, pulp water content, seed water content, and soluble solids content (SSC) were obtained according to the method of Yang et al. [39]. The soluble sugar content was measure using the anthrone colorimetric method [42]. The titratable acid was determined by following the protocol described by Yang et al. [39]. Three fruits were taken from each treatment, and the results were repeated three times.

### Statistical analysis

Data was recorded and calculated by Microsoft® Excel® 2021MSO (Redmond, Washington, USA). The data was subjected to one-way analysis of variance (ANOVA) implemented in SPSS 19.0 (Chicago, IL, USA, followed by Duncan’s new complex range method. We designated a *P*-value less than 0.05 as being a significant difference between means (*p* < 0.05). All figures were drawn using GraphPad Prism 6 (San Diego, CA, USA) and Adobe Illustrator CC2018 (Adobe, San Jose, CA, USA).

## Results

### Influence of bleeding on the anatomical structure of the leaf

**Fig. 1:**
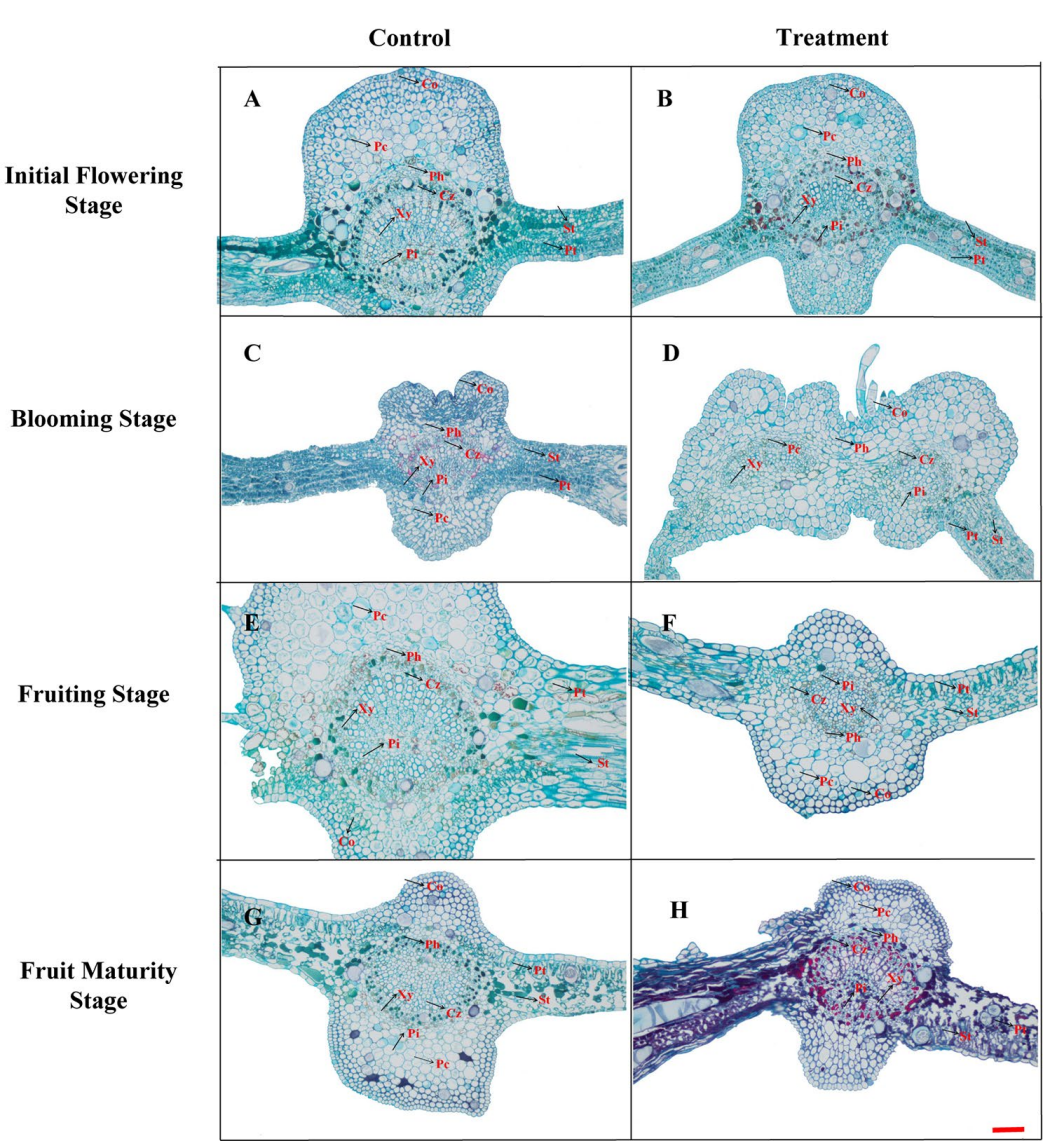
The anatomical structure of main veins of leaves Note: A-D: Control group; E-H: Treatment group; Xy: Xylem; Pc: Parenchyma cell; CZ: Cambium zone; Ph: Phloem; Pi: Pith; Co: Collenchyma; Pt: palisade tissue; St: Sponge tissue; Magnification: 100 times; Bar = 100 μm

We observed the leaf structure of *A. arguta* samples in detail, including collenchyma, palisade tissue, and sponge tissue (Fig. 1; Table 1). At the initial flowering stage, no noticeable differences in the anatomical structure of leaves were detected between the control and treatment (Fig. 1A, E). At the blooming stage, leaves in the treatment displayed a significantly greater number of collenchyma cells relative to that of the control (Fig. 1B, F), whereas the latter demonstrated a significantly increased thickness of xylem and phloem. At the fruiting stage, the control demonstrated an increased thickness of xylem and more regular arrangement of veins in comparison to that of the treatment (Fig. 1C, G). At the fruit maturity stage, the xylem of leaves in the treatment had an incomplete and damaged structure of xylem relative to control (Fig. 1D, H).Furthermore, the xylem and mesophyll integrity of the treatment was relatively poor., whereas the control harbored more collenchyma and parenchyma cells. These results confirmed that bleeding clearly damaged the leaf structure and variously affected the leaf development in different growth periods of *A. arguta* plants.

**Table 1:** Changes of leaf tissues in different periods

Period	Group	Xy/µm	Pc/µm	CZ/µm	Ph/µm	Pi/µm	Co/µm	Pt/µm	St/µm
Initial Flowering Stage	Control	27.67 ± 0.60a	71.30 ± 1.15a	18.00 ± 0.10a	15.00 ± 0.86b	15.33 ± 0.12a	34.67 ± 0.40a	16.00 ± 0.2a	21.67 ± 0.10b
	Treatment	12.67 ± 0.57b	62.67 ± 8.08b	17.00 ± 3.00a	18.67 ± 5.86a	14.00 ± 1.73a	27.00 ± 5.29b	15.33 ± 4.72a	26.67 ± 4.50a
Blooming Stage	Control	16.33 ± 2.31a	44.33 ± 4.04a	20.00 ± 1.00a	29.33 ± 7.51b	20.33 ± 4.04b	25.33 ± 8.10b	27.00 ± 5.56b	19.67 ± 4.16b
	Treatment	13.00 ± 2.64b	13.33 ± 0.57b	17.67 ± 4.73b	40.00 ± 8.72a	29.00 ± 0.00a	32.67 ± 2.89a	38.00 ± 4.00a	25.00 ± 2.64a
Fruiting Stage	Control	30.00 ± 8.66a	116.33 ± 10.69a	19.33 ± 4.16b	25.67 ± 4.04a	23.00 ± 4.36a	34.00 ± 11.35b	71.00 ± 10.00a	33.00 ± 3.46a
	Treatment	15.33 ± 1.52b	67.00 ± 8.54b	28.00 ± 4.00a	23.00 ± 2.00a	13.67 ± 3.21b	39.67 ± 8.08a	25.33 ± 8.32b	25.00 ± 4.35b
Fruit Maturity Stage	Control	18.67 ± 4.16a	45.00 ± 5.00a	28.67 ± 4.73a	20.00 ± 4.58b	50.33 ± 6.81a	21.67 ± 4.16a	47.67 ± 2.51a	25.67 ± 1.15a
	Treatment	15.67 ± 1.52b	36.67 ± 3.78b	20.00 ± 0.00b	26.00 ± 1.73a	16.00 ± 0.00b	19.00 ± 0.00a	46.67 ± 8.08a	22.33 ± 2.31b

Different letters indicate significant differences (*P* < 0.05)

Note: Xy: Xylem; Pc: Parenchyma cell; CZ: Cambium zone; Ph: Phloem; Pi: Pith; Co: Collenchyma; Pt: palisade tissue; St: Sponge tissue

### Effect of bleeding on leaf morphology and growth parameters

**Fig. 2:**
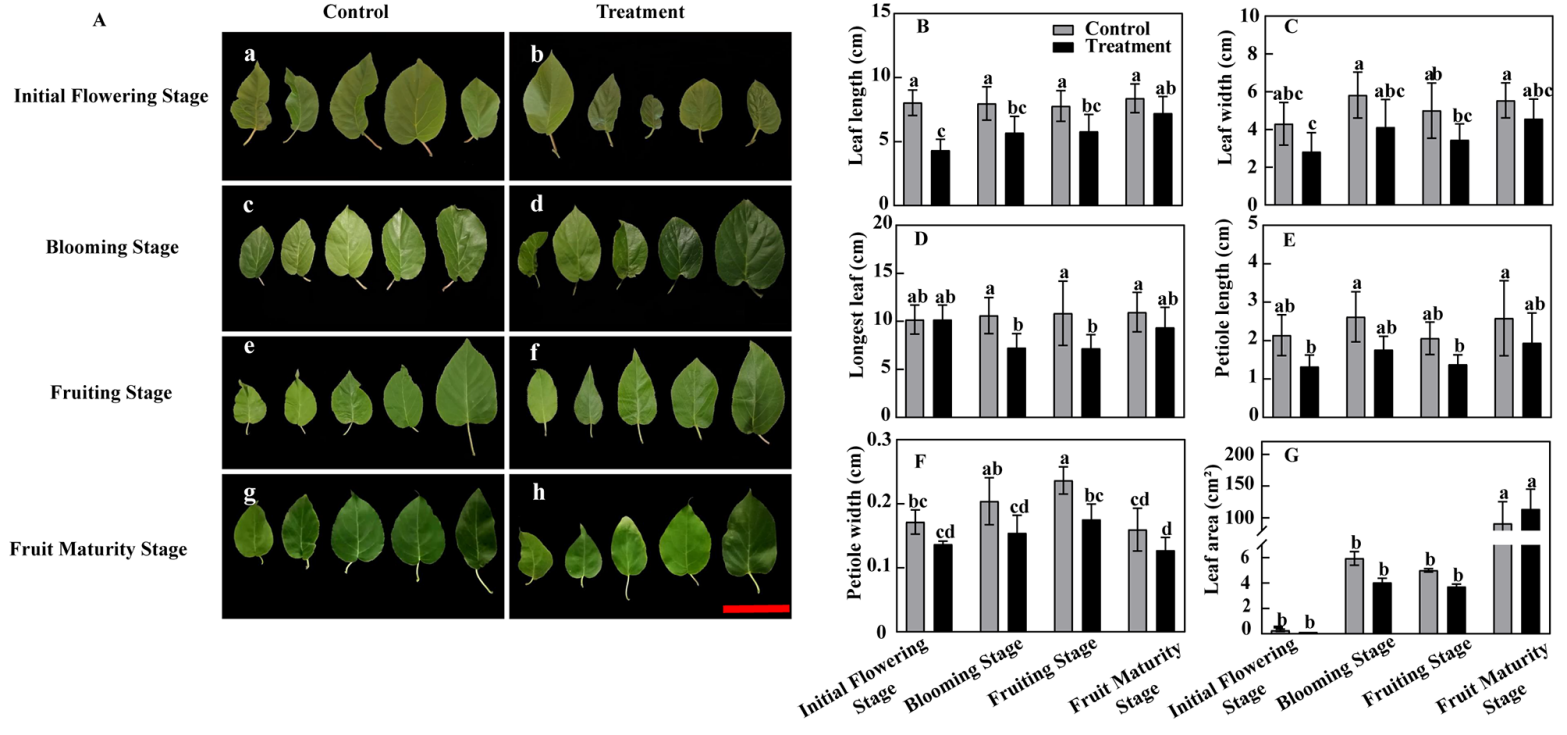
The effects of bleeding on leaves morphology and growth parameters in different phenological stages Note: A: a, c, e, g: Control group; b, d, f, h: Treatment group B: Leaf length; C: Leaf width; D: Petiole long; E: Petiole width; F: The longest leaf; G: Leaf area; Bar = 10 cm; Different letters indicate significant differences (*P* < 0.05)

There were significantly differences in leaf length, leaf width, longest leaf, petiole length, petiole width and leaf area between the control and treatment in different growth periods. The values of these parameters were generally lower in treatment than the control. Moreover, in any period, the leaves of control and treatment groups differed significant (Fig. 2B-G). In terms of their size, leaves in the treatment were significantly smaller than those in the control. In terms of their shape, the leaves in treatment were more deformed than those in the control. With the extension of the growth time, the leaf color was darker in the control than the treatment group (Fig. 2A). In addition, the number of leaf veins in the control significantly exceeded that in treatment. At the initial flowering stage, however, leaf length and leaf area were similar between the groups. Finally, at the blooming stage, fruiting stage, and fruit maturity stage, the leaf length and leaf area were smaller in the treatment than the control (Fig. 2B, G).

### Effects of bleeding on relative electrical conductivity, RWC and MDA content of leaves

**Fig. 3:**
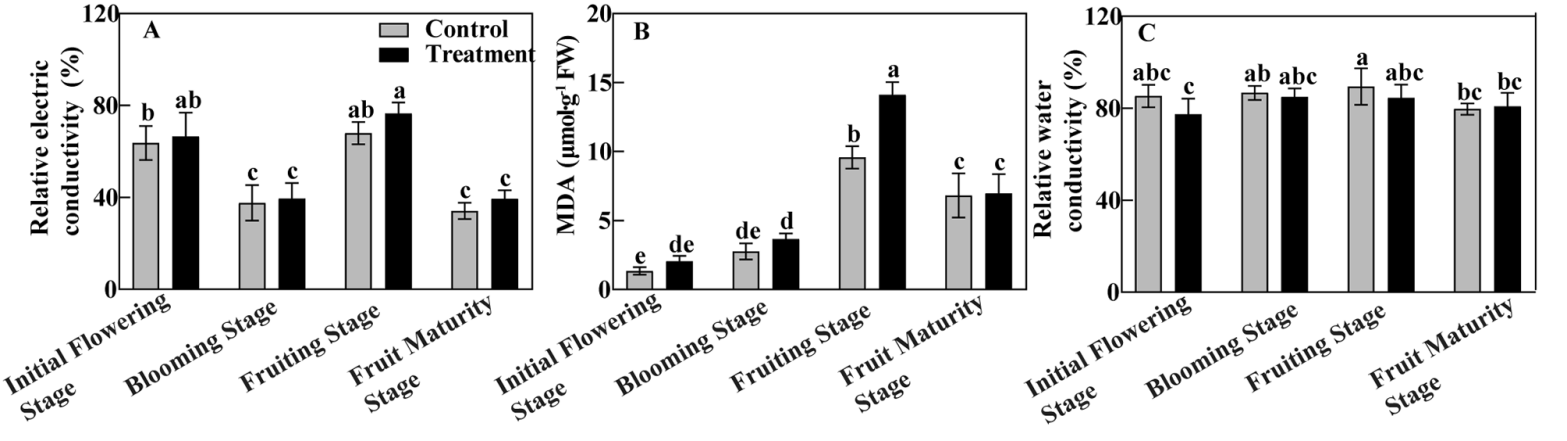
The effects of bleeding treatment on relative conductivity (**A**), relative water content (**B**) and malondialdehyde (MDA) content (**C**) of leaves at different phenological stages Note: A: Relative conductivity; B: Relative moisture content; C: Malondialdehyde; Different letters indicate significant differences (*P* < 0.05)

Relative electric conductivity in response to the treatment showed a trend of increasing from the initial flowering stage to fruit maturity stage (Fig. 3A). Compared with the control, the treatment had slightly higher relative electric values, indicating that the leaf tissue membrane was damaged. The maximum values of relative electric conductivity appeared at the fruiting stage and fruit maturity stage, respectively. Furthermore, the MDA contents of the leaves increased at first and then decreased across different phenology periods (Fig. 3B). At the initial flowering stage, blooming stage, fruiting stage and fruit maturity stage, the MDA content of leaves in the treatment exceeded that of the control, being significantly different at the fruiting stage, when the maximum MDA content of their leaves was reached: 10.36 µmol·g^− 1^ FW and 14.94 µmol·g^− 1^ FW, respectively. The RWC increased initially and then decreased (Fig. 3C), reaching its maximum value at the fruiting stage. In sum, bleeding can decrease the leaf water content and increase both the relative electric conductivity and MDA content of leaves, and at the fruiting stage this leaf damage was the greatest.

### Effects of bleeding current on chlorophyll content and photosynthetic parameters of leaves

**Fig. 4:**
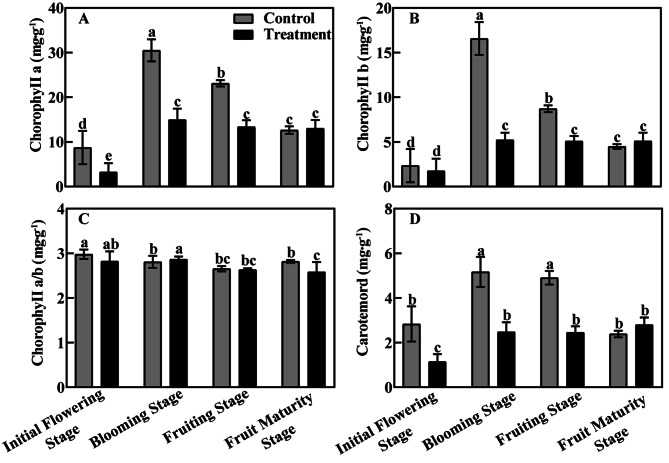
The effect of bleeding treatment on chlorophyll at different phenological periods Note: A: Chlorophyll a; b: Chlorophyll b; C: Chlorophyll a/b; D: Carotenoids; Different letters indicate significant differences (*P* < 0.05)

From the initial flowering stage to fruiting stage, the concentration of chlorophyll a as well as chlorophyll b in the leaves of the treatment group remained at a markedly lower level than those of the control group. However, in the fruit maturity stage the leaf chlorophyll a and chlorophyll b concentrations where higher in the treatment than the control (Fig. 4A, B). when further compared with the control, the ratio of chlorophyll a/b was lower in the treatment at the initial flowering stage, fruiting stage and fruit maturity stage but higher at blooming stag (Fig. 4C). The concentration of carotenoids generally decreased from initial flowering stage through fruiting stage, and then increased at fruit maturity stage (Fig. 4D).

**Fig. 5:**
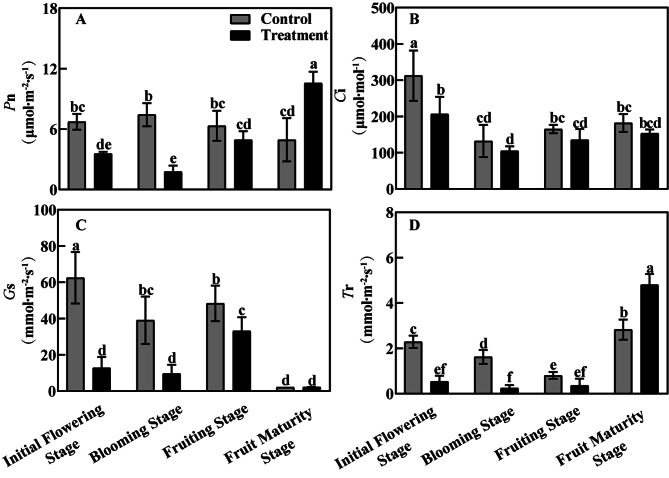
The effect of bleeding treatment on photosynthetic parameters of leaves in different phenological periods Note: A: The net photosynthetic rate (*P*n); B: Intercellular CO_2_ concentration (*C*i); C: Stomatal conductance (*G*s); D: Transpiration rate (*T*r); Different letters indicate significant differences (*P* < 0.05)

To further confirm that bleeding is responsible for changes in photosynthetic parameters, the values of *P*n, *C*i, *G*s and *T*r were determined for leaves in control versus treatment (Fig. 5). From initial flowering stage to fruiting stage, the *P*n, *C*i, *G*s and *T*r were higher in the control than the treatment. At the fruit maturity stage, however, the control group had lower values of *P*n and *T*r in the treatment, but the bleeding of *A*. *arguta* plants did not produce a significantly affect their *G*s.

### Effects of bleeding on ROS accumulates in leaves of *A*. *arguta*

**Fig. 6:**
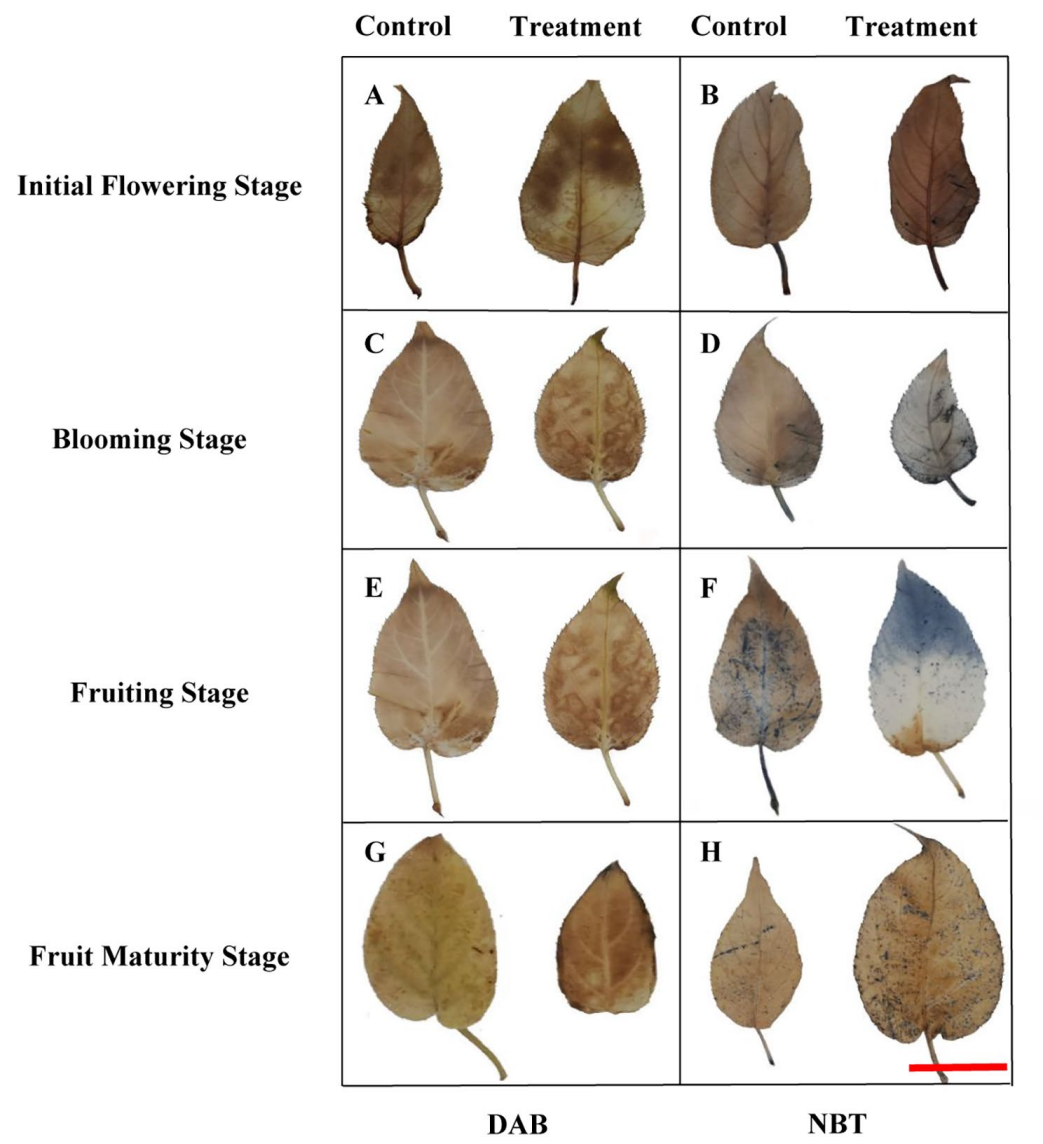
The effect of bleeding on DAB and NBT staining of leaves Note: A, C, E, G: DAB staining; B, D, F, H: NBT staining; Bar = 5 cm

Total H_2_O_2_ and O_2_^·−^ levels of leaves were examined in control and treatment groups. As clearly shown in Fig. 6, the NBT staining appeared lighter for the control than the treatment, being deepest at the fruiting stage. Further, O_2_^·−^ accumulation was enhanced in the treatment when compared with the control from the initial flowering stage to fruit maturity stage. DAB staining results showed that H_2_O_2_ content was significantly lower in the control relative to the treatment during all four growth stages.

### Effects of bleeding on fruit morphology, growth parameters and fruit quality

**Fig. 7:**
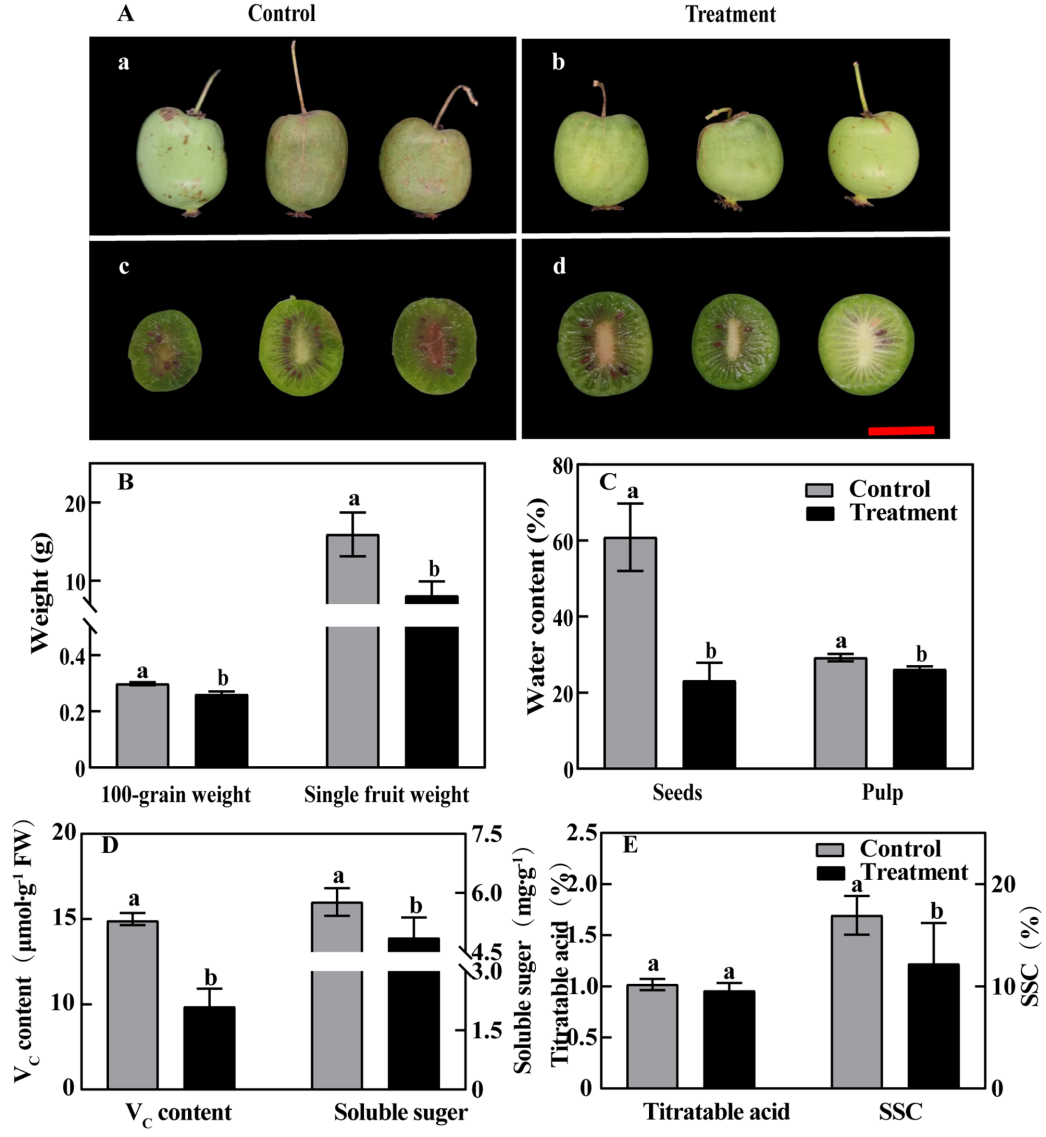
The fruit phenotypic image, phenotypic index and fruit quality during fruit ripening under bleeding condition Note: A: (a, c): Control group; (b, d): Treatment group; B: The 100-grain weight and single fruit weight; C: Seed water content and pulp water content; D: Vitamin C and soluble sugar; E: Titratable acid and soluble solids content (SSC); Bar = 5 cm; Different letters indicate significant differences (*P* < 0.05)

As Fig. 7A shows, the fruit of control had high maturity, full seeds and more seeds. In treatment, the fruit maturity was low, the phenomenon of late ripening occurred, and the seed development was incomplete. Also, the color of the peel was lighter after the treatment, whose fruit also appeared slight deformed. Thus, bleeding can lead to delayed fruit development, incomplete seed development, fruit deformity and other phenomenon. There was also significant difference between the control and treatment in terms of their 100-grain weight, single fruit weight, seed water content, and fruit fresh water content (Fig. 7B, C). The treatment group had a lower 100-grain weight (0.26 g), single fruit weight (8.07 g), seeds water content (23.19%) and pulp water content (26.18%).

The fruit of bled plants also had a reduced V_C_ content, soluble sugar content, titratable acid content and soluble sugar content (SSC). The fruit content of V_C_ in control was about twice that in the treatment and the soluble sugar content was higher in the control (5.82 mg·g^− 1^)than that in the treatment (4.04 mg·g^− 1^). The fruit titratable acid content was similar between the control and treatment, and the SSC of control was slightly higher than that of treatment, but the difference was small (Fig. 7D, E). The results showed a significant difference in fruit SSC between the control and treatment at 17.6% and 12.3%, respectively. In sum, bleeding resulted in substantial effects on the fruit quality of *A*. *arguta*.

## Discussion

It is well established that the medicinal value of plants’ bleeding sap is quite high [28]. However, such bleeding can induce the fruit canker disease during the production of plants. For instance, in European beech, the production of phytophthora increased the damage of bleeding to the tree body [11]. It was also found that bleeding canker is a devastating disease in the pear trees [43]. Therefore, during the process of collecting bleeding sap, care should be taken to avoid harming the tree body when bleeding it, and proper methods should be adopted to obtain high-purity bleeding sap [24]. In our study, the collection device was based on a celine bottle (Fig. S1); it seals well and reduces the risk of infection from pathogenic bacteria.

Although the bleeding sap itself has many benefits, bleeding can cause damage to *A*. *arguta*. We found that bleeding caused leaf deformities, increased the collenchyma tissues, damaged xylem and phloem integrity, and reduced the leaf area of *A*. *arguta*. Furthermore, the flowering period was delayed and shorter, the number of produced fruits decreased, and the immaturity rate increased. Accordingly, under this bleeding stress, the cell membrane first suffers functional damage as well as structural damage, leading to the extravasation of electrolytes in the cell, thereby augmenting the relative electrical conductivity [44, 45]. Hence, the degree of damage to cell membranes and the tolerance of plants can be gauged according to their changed level of relative electrical conductivity [46]. At different phenology stages of plants, the relative electrical conductivity of leaves in treatment was slightly higher than that in the control, indicating that the leaf’s tissue membrane is little damaged after bleeding. Besides, RWC is a reliable indicator of a plant’s water deficit and can be used to detect its response to that stress [47]. In our study, evidently the RWC of leaves decreased after bleeding. Furthermore, soluble sugars can operate as an osmotic regulator, which is conducive to the maintaining cell structure and functioning under stress conditions, and promotes the accumulation of proline to increase membrane stability [48, 49]. By examining various leaf physiological indices, it was also found that the content of MDA increased in content from the initial flowering stage to the fruiting stage, whereas in the fruit maturity stage, most of the physiological indices were reduced; this indicating that bleeding induces an stress response in *A. arguta* plants, a finding similar to that of Lu et al. [50]. In our study, the consistent pattern of changes in MDA and other osmotic regulatory substances may have been caused by the serious injury and loss of nutrients in treated plants, and may also be related to how they generally respond adversity while under stress. In this regard, this finding is consistent with a previous report on grapevine leaves, whose stress-related osmotic regulatory substances and stress products displayed upward trend, and the activity of protective enzymes also increased to a certain extent [18]. Thus, we propose that the bleeding phenomenon has a negative effect on plant growth.

In addition, the regulation of photosynthesis in response to environmental stimuli is a crucial process in how plants adapt to a given stress [51]. Photosynthesis is the basis of plant growth and development and is a pivotal factor determining productivity; as such, it is an important reference index in the research domains of plant cultivation, plant resistance to stress, and plant breeding. *P*n, *C*i, *G*s, and *T*r are crucial indicators reflecting the photosynthetic capacity of plant leaves and chlorophyll is the main pigment for photosynthesis in plants species [52–54]. Carotenoids have a strong antioxidant effect, and carotenoids were significantly lower in the bled plants than the control, indicating a reduced antioxidant capacity of plants due to the bleeding treatment [55]. Further, the chlorophyll a/b of leaves was lower in this treatment than that the control, which demonstrates that the cell membrane of plant leaves is damaged via bleeding and its ion exchange ability is weakened [56, 57]. Several studies have shown that the photosynthetic pigment and photosynthase contents influence the photosynthetic rate to a certain extent [58, 59], which is the consistent with our result. Hou et al. also suggested that the photosynthetic rate is positively correlated with the relative content of chlorophyll, and is closely related to *P*n, *T*r and *C*s. Notably, *P*n can reflect the *T*r and *C*s levels to a certain extent [60]. *P*n was the same with the overall trend that the contents of chlorophyll a, chlorophyll b and carotenoid were lower than that in control. This suggests that *P*n and chlorophyll content are positively correlated before and after the bleeding. This could be due to leaf deformities arising in the bleeding treatment as found in the present study, which affected the chloroplast functioning.

Increased production of ROS is a common consequence of exposure to some abiotic stresses. This arises from the excitation of the light reactions in photosynthesis [61]. Above we proved that stress from bleeding is capable inhibiting the photosynthetic process. Moreover, excessive accumulation of ROS can cause damage to plants, and this adversity can lead to oxidative bursts that increase ROS levels and cause cell damage [37]. From initial flowering stage to fruit maturity stage, the accumulation of O_2_^·−^ in the treatment was enhanced relative to the control. During all growth stages, more of H_2_O_2_ accumulates in the treatment, suggesting that the bleeding can increase ROS levels in *A*. *arguta* leaves.

The *A*. *arguta* is not only rich in nutrients, but also has high medicinal value [62]. In our study, it was found that the water content of fruit pulp decreased, and the water content of seeds differed greatly whether plants were bled or not. Likewise, the 100-seed weight and water content were lower after bleeding, but we could not determine whether that was due to the immature fruit or the fruit itself having fewer seeds, so further research was still needed. The Vitamin C (V_C_) content, soluble sugar content, and SSC in the fruit also decreased in bled plants. The SSC is also a key factor which sways consumer’s preferences, while V_C_ is an essential vitamins to maintain the normal physiological function of human body, which is mainly taken from fruits and the *A*. *arguta* is arguably rich in V_C_ [63]. So bleeding greatly influences on the quality of its fruit. Above, we speculated the leaf and fruit parameters’ change were mainly caused by bleeding.

On the whole, bleeding caused a delay in flowering and reduced both the germination rate and leaf area of *A. arguta* plants. Bleeding is also know to restrain the expression of flowering positive regulatory genes, such as *VvFLY* [64]. Bleeding sap can also promote plant growth and development to resist pathogen attack; for example, *Vvchi31* and *Vvchi17* play an pivotal role in postharvest disease-resistance of tomato and strawberry, respectively [65]. The NO^3−^ concentration in xylem when bleeding sap can reflect the drought resistance of maize [66], and root-bleeding sap contributed to learned root behavior, especially for improving nutrient and water uptake [25]. *A*. *arguta* has high vitality and strong climbing ability, it can be used for shading in summer and has landscape and ecological values [67]. Its fruit is a nutritious edible health fruit, but the fruit faces problems, such as a short harvesting time and poor storage resistance, so improving its storage potential and preservation technology is likely a key domain of future research and development [68]. Further, *A*. *arguta* is rich in wild resources. In recent years, the research and development of new varieties of *A*. *arguta* has been strengthened. Yet process in the *A*. *arguta* industry remains slow, and the speed of new varieties research should be accelerated [69]. Here, we only analyzed the influence of bleeding of the *A*. *arguta* ‘Kuilv’ variety; other varieties should be investigated, while the development and utilization of ‘Kuilv’ bleeding sap itself warrants further study. In the future, we plan to determine what its in the bleeding sap is composed of, to specify what went missing from the plant after the bleeding it, via gene expression analyses. In this way, we hope to elucidate the molecular mechanism of bleeding in *A*. *arguta.*

## Conclusion

Outcomes of the current study revealed that bleeding exerts a profound significantly negative impact on *A*. *arguta’s* growth, photosynthesis and chlorophyll content, and other physiological variables. Furthermore, bleeding increased the relative electrical conductivity and MDA and ROS production. The pulp and the seed water content of fruit and its vitamin C, soluble sugar content, and SSC decreased after the damage from bleeding. Serious bleeding will adversely affect the growth and development of *A. arguta* and fruit quality: slowing plant growth, wilting leaves, and reducing fruit yield. Then the plant is easy to infection and cause diseases and pests. Although it is difficult to avoid, the serious impact of bleeding on the plant deserves attention from farmers, who is aware of it could intervene; such as by pruning branches in time after leaves are shed to avoid too many or too heavy branches. Therefore, this study provides a theoretical basis for the physiological changes of *A*. *arguta* after bleeding, and can facilitate future research into *A*. *arguta* breeding strategies. Long-term field studies should be conducted to gain insights into the underlying molecular mechanisms.

### Electronic supplementary material

Below is the link to the electronic supplementary material.

Supplementary Material 1

## Data Availability

The datasets used and/or analyzed during the current study are available from the corresponding author on reasonable request.
