# Supplementary material for: Effects of bleeding of Actinidia arguta (Sieb. & Zucc) Planch. ex miq. on its plant growth, physiological characteristics and fruit quality
Source: BMC Plant Biol. 2023 Nov 2;23:531. doi: 10.1186/s12870-023-04560-w (PMC10621140; doi:10.1186/s12870-023-04560-w)
Supplement: Supplementary file 1 — Supplementary Material 1 [file 12870_2023_4560_MOESM1_ESM.docx]

**Supplemental material**


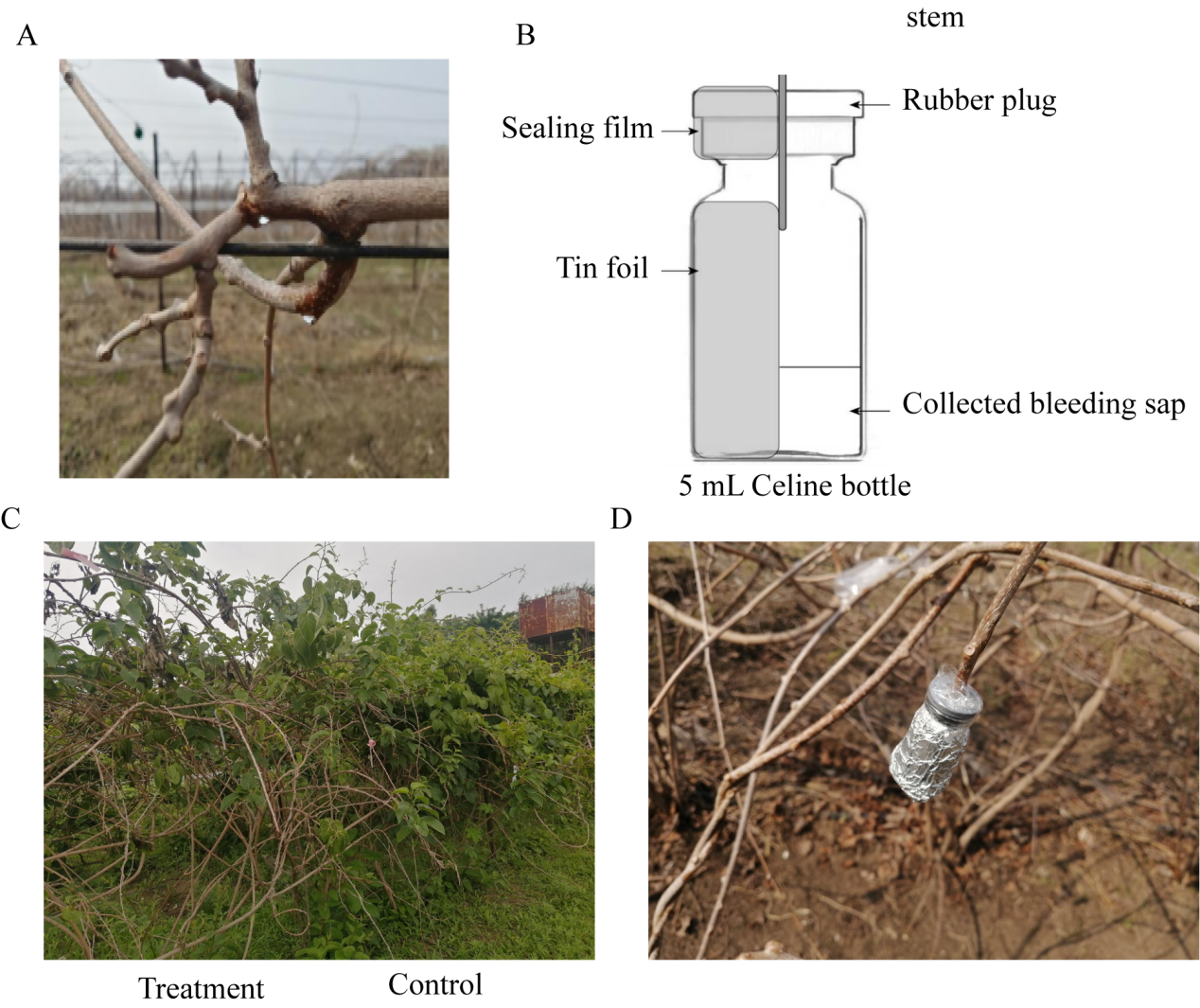


Fig. S1 The collection of bleeding and the photographs of field treatments

A: The bleeding treatment method; B: The specific structure of the bottle of celine; C: The field treatment; D: The collection of bleeding in field.
